# Clinical characteristics, pathogenic bacteria, and risk factors for different locations of pyogenic liver abscesses: a 10-year analysis

**DOI:** 10.3389/fcimb.2025.1692405

**Published:** 2026-01-07

**Authors:** JinHua Cui, YaMan Liu, Jian Li

**Affiliations:** 1 Department of Hepatobiliary Surgery,Affiliated Hospital of Chengde Medical College, Chengde, Hebei, China; 2 Department of Gynaecology, Affiliated Hospital of Chengde Medical College, Chengde, Hebei, China

**Keywords:** *Escherichia coli*, *Klebsiella pneumoniae*, left lobe of liver, pyogenic liver abscess, right lobe of liver

## Abstract

**Objective:**

This study aims to investigate the epidemiology, etiology, and clinical characteristics of patients with a pyogenic liver abscess treated at this institution over 10 years to offer insights into its clinical management.

**Methods:**

Patients diagnosed with a pyogenic liver abscess and hospitalized in the Affiliated Hospital of Chengde Medical College from June 2013 to June 2023 were retrospectively analyzed. The liver was categorized into left and right lobes using the middle hepatic vein as the boundary. Patients with abscesses encompassing both lobes were excluded. The patients were divided into left- and right-liver groups based on the abscess location. Epidemiological information, results of puncture drainage fluid or blood cultures, and drug sensitivity patterns were compared and analyzed between the groups. Furthermore, the clinical characteristics and treatment strategies for liver abscesses in different locations were summarized.

**Results:**

A total of 415 patients with pyogenic liver abscesses were included. Most cases (74.2%) encompassed abscesses in the right lobe. Gas was significantly more common within right-lobe abscesses than within left-lobe abscesses(*p*<0.05). Concerning pathogenic bacteria, the proportion of *Klebsiella pneumoniae* was significantly higher in right-lobe abscesses (44.5%,*p*<0.05) than in left-lobe abscesses. The proportion of *Escherichia coli* was significantly higher in left-lobe abscesses (10.3%,*p*<0.05). Previous abdominal surgery was considered a high-risk factor for *E. coli* infection in patients with left-lobe abscesses.

**Conclusions:**

*E. coli* accounted for a high proportion of left-lobe abscesses. Antimicrobial treatment with cefoperazone/sulbactam or piperacillin/tazobactam is recommended until pathogen culture findings are available, particularly for patients with liver abscesses with a history of abdominal surgery.

## 1 Introduction

A pyogenic liver abscess (PLA) is an intrahepatic infection caused by the invasion of pyogenic bacteria into the liver; it accounts for approximately 80% of all liver abscesses (Osman et al., 2018). The incidence of PLA has increased substantially in Asia. For example, in Taiwan, the annual incidence increased from 10.83 to 15.45 cases per 100,000 population between 2000 and 2011, with a mortality of 2% to 19%. This incidence is higher than that in Western countries (11.99 to 17.59 per 100,000 persons per year) (Cerwenka, 2010; Meddings et al., 2010; Chen et al., 2016). Numerous studies have highlighted diabetes mellitus, biliary tract disease, proton pump inhibitor use, prior abdominal surgery, and malignant tumors as major risk factors for PLA (Ho et al., 2017; Lin et al., 2017). Advancements in imaging technology have enabled conducting puncture catheter drainage, which has become the first-line treatment for PLA. When combined with antibiotic administration, puncture catheter drainage has significantly reduced PLA-related mortality (Cai et al., 2015; Ahmed et al., 2016; Tian et al., 2024). Researchers have investigated the influence of patient age or abscess size on the clinical characteristics of PLA (Kang and Hwang, 2011; Law and Li, 2013; Du et al., 2016). Nonetheless, no reports have demonstrated the influence of abscess location on PLA characteristics and etiology. Thus, this study seeks to compare the clinical profiles of patients with PLAs in the left and right lobes and to analyze the impact of abscess location on PLA diagnosis and treatment.

## 2 Materials and methods

### 2.1 Data collection

Clinical data of 504 patients diagnosed with PLA and hospitalized at the Affiliated Hospital of Chengde Medical College from June 2013 to June 2023 were retrospectively analyzed. The inclusion criteria were as follows: (i) PLA diagnosis confirmed by imaging examinations, such as computed tomography, magnetic resonance imaging, or ultrasound; (ii) characteristic PLA symptoms, such as fever and right upper abdominal pain; (iii) positive results from percutaneous transhepatic aspiration of purulent fluid; and (iv) bacterial confirmation from blood or pus culture. The exclusion criteria were as follows: (i) abscess at the junction of the left and right lobes or multiple abscesses occurring simultaneously; (ii) amebic or tuberculous liver abscess diagnosis. Finally, 415 patients with PLAs were analyzed.

The collected information comprised the following: demographic data (age, sex), clinical symptoms (fever, abdominal pain, vomiting, or fatigue), liver abscess characteristics (maximum diameter, whether separated, gas, and single or multiple abscesses); concomitant diseases (diabetes mellitus, hypertension, malignant tumor, history of abdominal surgery, gallstones, heart disease, or cerebral infarction); laboratory examinations (routine blood test, biochemistry, and coagulation), microbiological characteristics (pathogen culture rate, pathogen classification, and antibiotic sensitivity of common pathogens), treatment approach (percutaneous catheter drainage (PCD), percutaneous needle aspiration (PNA), antibiotics, and surgery), complications (septic shock, invasive syndrome); and patient prognosis (mortality and recurrence rates).in this study,invasive syndrome as the occurrence of distant metastatic infections due to hematogenous spread from the liver abscess, such as endophthalmitis, meningitis, brain abscess, pulmonary abscess or necrotizing fasciitis.

### 2.2 Ethical approval

The study protocol was approved by the Ethics Committee of the Affiliated Hospital of Chengde Medical College (CYFYLL2022507). It adhered to the ethical standards outlined in the 1964 Declaration of Helsinki and its subsequent amendments. Because of its retrospective design, the need for informed consent was waived.

#### 2.2.1 Treatment

All patients received antibiotic therapy. Empirical regimens, typically consisting of third-generation cephalosporins, β−lactam/β−lactamase inhibitor combinations, or fluoroquinolones, were initiated upon diagnosis and continued until pathogen identification and antimicrobial susceptibility testing results became available. If the empirically chosen antibiotic was included in the susceptibility profile, treatment was continued with the same agent. Otherwise, therapy was escalated or adjusted according to the sensitivity results, such as switching to cefoperazone/sulbactam, piperacillin/tazobactam, or carbapenems in cases of suspected or confirmed resistance. With informed consent, percutaneous catheter drainage (PCD) was performed for abscesses larger than 3 cm in diameter with adequate liquefaction, using an 8−to 10−French pigtail catheter. For abscesses smaller than 3 cm, percutaneous needle aspiration (PNA) was recommended.

#### 2.2.2 Statistical analysis

SPSS23.0 statistical software was used for all analyses. Descriptive statistics are presented as frequencies and percentages. Categorical variables were compared using the χ^2^ test, whereas continuous variables were compared using the t-test. Origin drawing software was used to plot antibiotic sensitivity data for pathogenic bacteria in the observation and control groups. Logistic regression analysis was conducted to assess the correlation between *Escherichia coli* as a causative pathogen of left-lobe abscesses and other factors. Only significant variables from the univariate analysis were included in the multivariate analysis.

## 3 Results

### 3.1 Demographic and clinical characteristics

Comparisons of various parameters are depicted in **Tables 1**, **2**. **Table 1** shows comparisons of demographic, clinical, and laboratory characteristics between patients with left- and right-lobe abscesses, while **Table 2** summarizes microbiological, therapeutic, and outcome comparisons.

**Table 1:** Demographics, clinical characteristics and laboratory examination of 415 PLA patients in the two groups.

Variables	Observation group (n=107)	Control group(n=308)	P-value
Gender(n,%)			
Male	69(64.5%)	201(65.3%)	0.885
Female	38(35.5%)	107(34.7%)	
Age(mean SD)	59.5±14.7	58.58±13.23	0.537
The size of PLA(mean mm)	64.7±27.2	67.6±24.9	0.323
Multiloculated PLA(n,%)	48(44.9%)	142(46.1%)	0.803
Contains gas(n,%)	5(5.7%)	36(11.7%)	0.036
Individual PLA(n,%)	99(92.5%)	258(83.8%)	0.024
Multiple PLA(n,%)	8(7.5%)	50(16.2%)	
Symptoms (n,%)			
Fever	95(88.8%)	282(91.6%)	0.391
Abdominal pain	59(55.1%)	155(50.3%)	0.391
Vomit	23(21.5%)	62(20.1%)	0.763
Weakness	68(63.6%)	211(68.5%)	0.347
Comorbidities(n,%)			
Diabetes	42(39.2%)	118(38.3%)	0.863
Hypertension	22(20.6%)	71(23.1%)	0.594
Malignant tumor	16(14.9%)	33(10.4%)	0.242
Abdominal operation	19(17.8%)	50(16.2%)	0.715
Cholecystolithiasis	23(21.5%)	56(18.2%)	0.452
Heart disease	17(15.9%)	30(9.7%)	0.08
Cerebral infarction	7(6.5%)	31(10.1%)	0.276
laboratory examination			
ALT (U/L)	59.0±35.9	62.8±44.4	0.363
FIB(g/L)	5.6±1.3	5.7±1.4	0.716
ALB(g/L)	32.3±5.4	32.2±5.1	0.800
PLT(×109/L)	206.3±150.9	228.6±139.4	0.165
PCT(ng/ml)	14.6±21.7	14.7±27.3	0.971
CRP(mg/L)	112.6±84.8	120.4±72.5	0.406
WBC(×109/L)	10.5±5.3	10.4±5.4	0.966

PLA, Pyogenic liver abscess; FIB, Fibrinogen; ALB, albumin; ALT, Alanine aminotransferase; WBC, White blood cell count PLT, Blood platelet; PCT, Procalcitonin; CRP, C-reactive protein.

**Table 2:** Pathogen species, treatment and complications of 415 PLA patients in the two groups.

Variables	Observation group(n=107)	Control group(n=308)	P-value
Pathogen culture positive	51(47.7%)	169(54.9%)	0.198
Klebsiella pneumoniae	34(31.8%)	137(44.5%)	0.017
E.coli	11(10.3%)	15(4.9%)	0.032
C. difficile	1(0.9%)	4(1.3%)	1.000
Streptococcus formata	3(2.8%)	8(2.6%)	0.746
Aerobacter cloacae	1(0.9%)	2(0.6%)	0.581
Bacaeroides fragilis	0(0 %)	1(0.3%)	1.000
Fungus	1(0.9%)	2(0.6%)	0.581
Invasive syndrome	7(6.5%)	13(4.2%)	0.334
Death	3(2.8%)	13(4.2%)	0.512
Septic shock	7(6.5%)	31(10.1%)	0.276
Recurred within six months	12(11.2%)	40(13%)	0.633
Admission to the ICU	8(7.4%)	32(10.3%)	0.379
Pleural effussion	42(39.2%)	103(33.4%)	0.291
Treatment methods			
PCD+Antibiotics	59(55.1%)	208(67.5%)	0.026
Antibiotics	46(42.9%)	90(29.2%)	0.012
Surgery+Antibiotics	0(0 %)	1(0.3%)	1.000
PNA+Antibiotics	2(1.8%)	9(2.9%)	0.736

PCD, percutaneous catheter drainage; ICU, intensive care unit; PNA, Puncture aspiration.

The sex, mean age at PLA onset, liver abscess size, septation or pneumatosis, and the number of liver abscesses did not differ between the two groups. Additionally, no significant differences were observed in the symptoms, including fever, abdominal pain, vomiting, and fatigue, between these groups (**Table 1**).

### 3.2 Concomitant diseases

The prevalence of concomitant diseases, including diabetes, hypertension, coronary heart disease, cerebral infarction, gallstone, abdominal surgery, malignant tumor, and chronic kidney disease, did not differ between the groups (**Table 1**).

### 3.3 Laboratory results

No significant differences were detected in infection biomarkers, including white blood cell count, procalcitonin, and C-reactive protein, between the two groups. Additionally, liver function indexes, including alanine aminotransferase, total bilirubin, and albumin levels, did not differ between these groups. No significant differences were detected in fibrinogen levels and platelet count between the groups (**Table 1**).

### 3.4 Pathogenic bacteria

The proportion of patients who tested positive for pathogenic bacteria did not differ between the left- and right-lobe abscess groups (47.7% vs. 54.9%,p=0.198, difference -7.2%; 95% CI -16.5% to 2.1%). Klebsiella pneumoniae and Escherichia coli were the most common pathogens in both groups. The proportion of E. coli was significantly higher in the left-lobe abscess group (10.3% vs. 4.9%,p= 0.032,difference 5.4%; 95% CI, 0.6% to 10.2%). However, the proportion of K. pneumoniae was significantly higher in the right-lobe abscess group (44.5% vs. 31.8%,p= 0.017, difference 12.7%; 95% CI, 2.4% to 23.0%) (**Table 2**).

### 3.5 Treatment and complications

Concerning treatment, the proportion of patients undergoing PCD combined with antibiotics was significantly lower in the left-lobe abscess group than in the right-lobe abscess group (55.1% vs. 67.5%,p= 0.026,difference -12.4%; 95% CI, -22.0% to -2.8%). Contrarily, the proportion of patients receiving antibiotic therapy alone was significantly higher in the left-lobe abscess group than in the right-lobe abscess group (42.9% vs. 29.2%,p= 0.012, difference 13.7%; 95% CI, 4.5% to 22.9%). However, no significant differences were observed in the rates of surgeries and PNA between the two groups. Regarding complications, no significant differences were observed in the incidence of septic shock, invasive syndrome, recurrence rate within 6 months, pleural effusion rate, intensive care unit admissions, and mortality between the two groups (**Table 2**).

### 3.6 Drug sensitivity test results

**Figure 1:**
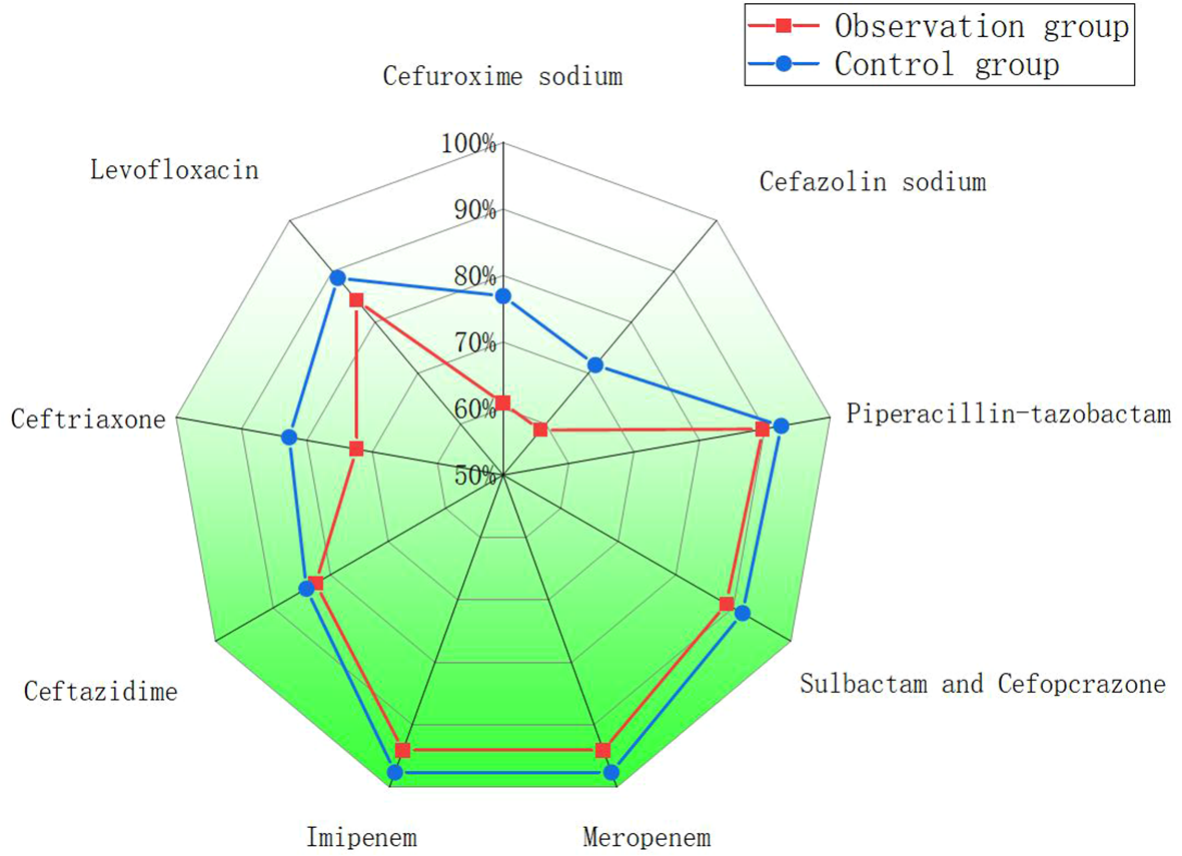
Comparison of antibiotic resistance in two groups of patients with PLA.

Drug sensitivity test results suggested that meropenem and imipenem displayed the highest sensitivity, followed by cefoperazone/sulbactam and piperacillin/tazobactam, with sensitivity rates reaching approximately 90%. Levofloxacin displayed a sensitivity exceeding 80% in both groups. Both pathogens displayed relatively poor sensitivity to other antibiotics (**Figure 1**).

### 3.7 High-risk factors for *E. coli* infection

Univariate and multivariate analysis results suggested that a history of abdominal surgery was a major risk factor for *E. coli* infection in patients with left-lobe abscesses (**Table 3**).

**Table 3:** Risk factors for *Escherichia coli*.

Variables	Univariate analysis	P-value	Multivariate analysis	P-value
	Odds ratio (95% CI)		Odds ratio (95% CI)	
History of abdominal surgery	4.800(1.163-19.805)	0.030	4.406(1.037-18.716)	0.045
diabetes	0.517(0.131-2.047)	0.347		
Gender	1.284(0.291-5.256)	0.741		
Age	1.041(0.985-1.100)	0.152	1.035(0.978-1.095)	0.232
The mean size of PLA	0.991(0.966-1.017)	0.477		
Cholecystolithiasis	2.286(0.535-9.767)	0.265		
Multiloculated PLA	1.200(0.315-4.578)	0.790		

PLA, Pyogenic liver abscess;

## 4 Discussion

In this study, bacterial liver abscesses were primarily located in the right liver lobe, consistent with previous reports (Yin et al., 2021). Fever was the primary symptom in both left- and right-lobe abscesses, with elevated levels of white blood cell count, procalcitonin, and C-reactive protein. Diabetes mellitus was the most common comorbidity in both groups, consistent with previous studies (Cui et al., 2023). Compared with left-lobe abscesses, the presence of gas was more associated with right-lobe abscesses. Bacterial liver abscesses containing gas pose a high risk of sepsis and prolonged hospitalization; they are common after biliary surgery (Zhang et al., 2020). Chou reported that multiple liver abscesses were frequently associated with biliary tract infections and resulted in higher mortality than a single liver abscess (Chou et al., 1997). This necessitates more aggressive treatment to reduce the risk of mortality.

Klebsiella pneumoniae was the most common pathogen in both left- and right-lobe abscesses, aligning with previous reports (Zhang et al., 2019; Chan et al., 2022). A novel finding of our study was the significantly higher proportion of Escherichia coli in left-lobe abscesses, a distinction that warrants further investigation. Consistent with Zhang et al (Zhang et al., 2018), multivariate analysis identified a history of abdominal surgery as an independent risk factor for E. coli infection. In our cohort, the most common prior surgeries were gallbladder/biliary procedures, followed by gastrectomy, colorectal resection, and pancreaticoduodenectomy, echoing the hepatobiliary-gastrointestinal surgical profile associated with PLA risk (Zhang et al., 2018). This supports the concept that anatomical alterations from such surgeries create a risk landscape favoring E. coli, as also seen in post-pancreaticoduodenectomy (Chen et al., 2019) and biliary surgery (Lin et al., 2024) patients. Given that E. coli liver abscesses carry a higher risk of septic shock and mortality (Ruiz-Hernandez et al., 2019), our finding underscores the need for heightened vigilance in managing left-lobe abscesses, which presented with a greater E. coli burden.

The treatment of left-lobe abscesses is less aggressive than that of right-lobe abscesses, probably because of the high risk of puncture and drainage caused by their proximity to the heart and gastrointestinal tract. Endoscopic ultrasound-guided drainage is considered a safe and effective intervention for left-lobe abscesses, without the risk of accidental tube detachment (Rana et al., 2020). Furthermore, open surgical drainage combined with antibiotic therapy has been considered a safe and effective treatment for left-lobe abscesses (Swartawan and Saputra, 2023). In this study, PNA was significantly less frequently conducted than PCD, attributed to the need for repeated PNA, which increases the risk of bleeding. Moreover, PCD exhibits superior therapeutic efficacy for large, separated abscesses (Kumar et al., 2019). PNA is superior to PCD because of its higher success rate, lower cost, shorter hospitalization, and reduced puncture pain, particularly for abscesses measuring 5 to 10 cm in diameter (Zhang et al., 2024). However, most studies favor PCD, highlighting its higher cure rate, faster recovery, and reduced duration of antibiotic therapy (Lin et al., 2023).

Bacterial culture results indicated that left-lobe abscesses demonstrated lower sensitivity to common antibiotics than right-lobe abscesses, particularly to second- and third-generation cephalosporins. This finding is probably attributed to the higher proportion of multidrug-resistant E. coli in left-lobe abscesses (Liu et al., 2023). In the present study, cefoperazone/sulbactam and piperacillin/tazobactam were strongly effective against PLA. Therefore, these antibiotics are recommended for patients newly diagnosed with PLA. Our empirical antibiotic practice evolved during the study period toward the preferential use of these β-lactam/β-lactamase inhibitor combinations as first-line therapy, particularly in light of the higher prevalence and resistance profile of E. coli observed in left-lobe abscesses. If the initial antibiotic therapy proves ineffective, carbapenems may be considered. This approach is consistent with previous recommendations that cefoperazone/sulbactam or piperacillin/tazobactam be used as first-line empirical treatments for PLA, especially before pathogen identification (Yang et al., 2024). E. coli is the primary pathogen implicated in recurrent liver abscesses (Cui et al., 2025). In this study, despite a higher proportion of E. coli in left-lobe liver abscesses, the recurrence rates did not differ between the groups. Additionally, no significant difference in mortality was observed between left- and right-lobe abscesses (Lee et al., 2021; Chan et al., 2022; Jiménez-Romero et al., 2023).

This study has certain limitations. Its single-center retrospective design may have introduced information bias. For instance, the negative rate of bacterial cultures in this study was excessively high, which may be attributed to the previous use of antibiotics.This warrants future multi-center studies with larger sample sizes to improve the accuracy of the results.

## Conclusion

*E. coli* was the primary pathogen in left-lobe PLA. Cefoperazone/sulbactam or piperacillin/tazobactam is recommended for antibiotic therapy until pathogenic culture results become available, particularly for patients with a history of abdominal surgery.

## Data Availability

The datasets presented in this study can be found in online repositories. The names of the repository/repositories and accession number(s) can be found below: https://doi.org/10.57760/sciencedb.22607.
